# Association between Different Indexations of Extravascular Lung Water (EVLW) and PaO_2_/FiO_2_: A Two-Center Study in 231 Patients

**DOI:** 10.1371/journal.pone.0103854

**Published:** 2014-08-05

**Authors:** Wolfgang Huber, Josef Höllthaler, Tibor Schuster, Andreas Umgelter, Michael Franzen, Bernd Saugel, Colin Cordemans, Roland M. Schmid, Manu L. N. G. Malbrain

**Affiliations:** 1 II. Medizinische Klinik und Poliklinik, Klinikum rechts der Isar der Technischen Universität München, München, Germany; 2 Institut für Medizinische Epidemiologie und Statistik, Klinikum rechts der Isar der Technischen Universität München, München, Germany; 3 Department of Intensive Care, Ziekenhuis Netwerk Antwerpen, Antwerpen, Belgium; Duke University Medical Center, United States of America

## Abstract

**Background:**

Variability of body weight (BW) and height calls for indexation of volumetric hemodynamic parameters. Extravascular lung water (EVLW) has formerly been indexed to *actual* BW (BW_act_) termed EVLW-index (EVLWI). In overweight patients indexation to BW_act_ might inappropriately lower indexed EVLWI_act_. Several studies suggest indexation of EVLWI to *predicted* BW (EVLWI_pred_). However, data regarding association of EVLWI_act_ and EVLW_pred_ to mortality and PaO_2_/FiO_2_ are inconsistent. Two recent studies based on biometric database-analyses suggest indexation of EVLWI to height (EVLWI_height_). Therefore, our study compared the association of un-indexed EVLW, EVLWI_height_, EVLW_pred_ and EVLWI_act_ to PaO_2_/FiO_2_ and Oxygenation index (OI = mean airway pressure*FiO_2_*/PaO_2_).

**Methods:**

A total of 2119 triplicate transpulmonary thermodilutions (TPTDs; PiCCO; Pulsion Medical-Systems, Germany) were performed in 50 patients from the evaluation, and 181 patients from the validation groups. Correlations of EVLW and EVLWI to PaO_2_/FiO_2_, OI and ROC-AUC-analyses regarding PaO_2_/FiO_2_<200 mmHg (primary endpoint) and OI>10 were performed.

**Results:**

In the evaluation group, un-indexed EVLW (AUC 0.758; 95%-CI: 0.637-0.880) and EVLWI_height_ (AUC 0.746; 95%-CI: 0.622-0.869) provided the largest ROC-AUCs regarding PaO_2_/FiO_2_<200 mmHg. The AUC for EVLWI_pred_ was smaller (0.713). EVLWI_act_ provided the smallest AUC (0.685). This was confirmed in the validation group: EVLWI_height_ provided the largest AUC (0.735), EVLWI_act_ (0.710) the smallest. In the merged data-pool, AUC was significantly greater for EVLWI_height_ (0.729; 95%-CI: 0.674–0.784) compared to all other indexations including EVLWI_act_ (ROC-AUC 0.683, p = 0.007) and EVLWI_pred_ (ROC-AUC 0.707, p = 0.015). The association of EVLW(I) was even stronger to OI compared to PaO_2_/FiO_2_. In the merged data-pool, EVLWI_height_ provided the largest AUC regarding “OI>10” (0.778; 95%-CI: 0.713–0.842) compared to 0.739 (95%-CI: 0.669–0.810) for EVLWI_act_ and 0.756 (95%-CI: 0.688–0.824) for EVLWI_pred_.

**Conclusions:**

Indexation of EVLW to height (EVLWI_height_) improves the association of EVLW(I) to PaO_2_/FiO_2_ and OI compared to all other indexations including EVLWI_pred_ and EVLWI_act_. Also considering two recent biometric database analyses, EVLWI should be indexed to height.

## Introduction

Extravascular lung water (EVLW) is a measure of the interstitial, alveolar and lymphatic fluid content of the lungs. EVLW and its indexation to body weight (EVLWI) became routinely available after the introduction of single-indicator trans-pulmonary thermodilution (TPTD) [1]–[5]. A number of animal and clinical studies demonstrated an association of EVLW(I) to mortality and to parameters of pulmonary function such as PaO_2_/FiO_2_ [5]–[19]. Variability of body weight (BW) and height strongly calls for biometric adjustment and indexation. *Ideally*, appropriate indexation should be based on a limited number of routinely available biometric data, and it should result in consistent normal values for patients with different height, weight, age, gender and race [20]–[21]. Originally actual body weight (BW_act_) was used for indexation of EVLW. However, triggered by a rapidly increasing number of obese patients [22], [ 23], the question arose as to *which* weight to choose for EVLW indexation since indexation to BW_act_ might inappropriately decrease EVLWI_act_ in obese patients. Based on a better correlation to mortality, a number of studies have suggested indexation of EVLW to predicted BW (EVLWI_pred_) (see Table 1) [12: 14]. However, data are inconsistent regarding other endpoints: e.g. if available data provide worse [12], similar [14], [ 16] or slightly better correlation of EVLWI_pred_ to PaO_2_/FiO_2_ than EVLWI_act_ [13]. In one of the most recent studies [15], “Chew et al. found that EVLW indexed to absolute body weight resulted in a stronger association with outcome” including mortality compared to EVLWI_pred_ [24]. Despite the overall strong predictive capacity of EVLWI in this and other recent trials [12]–[19], these inconsistencies demonstrate the need to optimize indexation of EVLW. Therefore, we recently analyzed a prospectively maintained database regarding the association of EVLW to biometric data [18]. This study demonstrated that height was the only biometric parameter independently associated to EVLW. These data were recently confirmed by Wolf et al., using a similar approach in a surgical group [19]. Despite these conclusive data, both studies did not investigate, if indexation of EVLW to height (EVLWI_height_) provides better association to pulmonary function and outcome. Furthermore, all these trials were mono-centric.

**Table 1:** Indexations of extravascular lung water (index) EVLW/EVLWI.

EVLWI_act_ = EVLW/BW_act_		
ELWI_pred_ = EVLW/BW_pred_	BW_pred_ = Predicted body weight [kg]:	Male: 50+0.91×(height – 152.4)
		Female: 45.5+0.91×(height – 152.4)
EVLWI_id_ = EVLW/BW_id_	BW_id_ = Ideal body weight [kg]:	Male: (height – 100)×0.9
		Female: (height – 100)×0.85
EVLWI_adj_ = EVLW/BW_adj_	Adjusted body weight [kg]:	Male: ideal_male_BW + (actual BW – ideal_male_BW)×0.4
		Female: ideal_female_BW + (actual BW – ideal_female_BW)×0.4
EVLWI_height_ = EVLW/height [cm]		
EVLWI_BMI_ = EVLWI/BMI	BMI = Body Mass Index [kg/m^2^]:	BW_act_ [kg]/(height[m])^2^
EVLWI_BSA_ = EVLW/BSA_Dubois_	BSA_Dubois_ [m^2^] = 0.007184*weight [kg]^0.425^*height [cm]^0.725^	
EVLWI_TLC_ = EVLW/TLC	TLC = Total Lung Capacity TLC [L]:	Male: 7.99*height [m] –7.08
		Female: 6.60*height [m] – 5.79

Therefore, this two-center study compared the association of PaO_2_/FiO_2_ (and other outcome markers) to EVLW (un-indexed), EVLWI_act_, EVLWI_pred_, EVLWI_height_ and EVLWI indexed to other biometric indices. To provide sufficient balance of biometric data, we investigated a group with a representative distribution of body mass index (BMI) [22], as well as an unselected second group from a second center.

## Materials and Methods

### Munich-evaluation-group

The institutional ethics committee approved the study (Ethikkommission Technische Universität München; Fakultät für Medizin; No. 3049/11). Patients on mechanical ventilation monitored using TPTD regardless of the study were included in the prospectively maintained database. The need for informed consent was waived due to the non-interventional design of the study. The patients included in this study completely distinct to the group previously analysed regarding the association of EVLW to biometric data [18].

To provide a representative distribution regarding bodyweight [22], we included 15 consecutive patients with BMI≥30 kg/m^2^, 15 consecutive patients with 25≤BMI<30 kg/m^2^ and 20 consecutive patients with a normal BMI (<25 kg/m^2^) irrespective of fulfilling the criteria of acute respiratory distress syndrome (ARDS). Conscious patients were asked for actual biometric data. In unconscious patients body weight and height were extracted from the patients records. In case of doubt height was verified using a flexible tape measure in the supine position.

A 5-F thermistor-tipped femoral arterial line (PV2025L20, Pulsiocath, Pulsion Medical Systems, Munich, Germany) connected to the PiCCO monitor device (PiCCO-Plus; Pulsion Medical Systems) was used for TPTD measurements. The mean EVLW was measured based on TPTD performed in triplicate with 15 ml cold saline 0.9%.

### Antwerp-validation-group

Retrospective analysis of data from a prospectively developed independent cohort was performed for the first 7 days of ICU admission. Data of 181 critically ill patients requiring mechanical ventilation and TPTD-hemodynamic monitoring treated in two ICU's in ZNA Campus Stuivenberg, Antwerp, Belgium were collected prospectively. Ethics approval had been obtained and due to the retrospective analysis and non-intervention-nature of the study the need for informed consent was waived (project number EC 3765; Commisie voor Medische Ethiek, Ziekenhuisnetwerk Antwerpen 2020). Parts of the data not related to EVLW-indexation have already been published in Annals of Intensive Care [25], [ 26].

The measuring technique was identical to the Munich-evaluation-group, with the only difference being that three 20 ml boluses of cooled saline were used for TPTD.

Mean length of the ICU-stay in the Munich group was 27.2±21.4 days with a range of 3 to 120 days. In the Antwerp group the mean ICU stay was 25.9±41.7 days with a range of 1to 429 days.

### Endpoints and Statistics

**Table 2:** Patients' characteristics.

	Munich-Evaluation-Group								Antwerp-Validation-Group	
	**All patients**		**BMI<25 kg/m^2^**		**25≤BMI<30 kg/m^2^**		**BMI≥30 kg/m^2^**		**All patients from Antwerp**	
**Gender**	22 female, 28 male		6 female, 14 male		7 female, 8 male		9 female, 6 male		62 female, 119 male	
**Age**	64.1±14.8 years		67.6±14.3		62.5±18.7		63.3±11.2		62.4±16.4	
**Weight**	82.0±23.5		63.9±9.2		78.7±12.2		105.1±29.2		74.5±17.6	
**Height**	169±10.1		169±9.0		172±13.0		169±8.9		170.1±11.6	
**Body mass index**	28.3±6.7		22.0±1.9		27.4±1.3		35.9±6.3		25.6±5.5	
**APACHE II-Score**	24.9±9.8		25.7±8.6		23.2±7.8		24.6±9.2		22.9±10.3	
**Etiology**									Respiratory failure:	
Sepsis	21		11		3		7		- acute on chronic 11/181	
Pneumonia/ARDS	11		3		3		5		- acute 152/181	
Liver cirrhosis	7		3		3		1		- coma 18/181	
Pancreatitis	4		1		2		1		Medical patients 158	
Other etiology	7		2		4		1		Surgical patients 23	
**Parameter**	**Mean ± SD**	**Median; COV**	**Mean ± SD**	**Median; COV**	**Mean ± SD**	**Median; COV**	**Mean ± SD**	**Median; COV**	**Mean ± SD**	**Median; COV**
**EVLWI unindex.**	716±363	637; 0.51	711±266	700; 0.37	618±270	560;0.44	783±469^§^	660; 0.60	773±346	683; 0.45
**EVLWI_act_**	8.9±3.8	8; 0.43	11.2±3.7	11; 0.33	7.7±3.1	7; 0.40	7.4±3.0^‡^	6; 0.41	10.6±4.7	10; 0.44
**EVLWI_pred_**	10.8±5.2†	9; 0.48	10.7±3.9†	10; 0.36	9.3±4.4†	8; 0.47	11.7±6.3†^§^	10; 0.54	11.9±5.7†	11; 0.48
**EVLWI_id_**	11.2±5.4†	10; 0.48	11.2±4.1	11; 0.37	9.6±4.5†	8; 0.47	12.3±6.6†^§^	10; 0.54	12.3±5.9†	11; 0.48
**EVLWI_ad_**	9.7±4.2†	9; 0.43	11.0±3.8†	11; 0.35	8.4±3.8†	7; 0.45	9.3±4.3†^‡^	8; 0.46	11.4±5.5†	10; 0.48
**EVLWI_BMI_**	26.3±11.9	24.2; 0.45	32.9±11.7	31.4; 0.36	22.8±9.8	21.0; 0.43	22.0±10.4^‡^	20.0; 0.47	30.6±13.3	28.2; 0.43
**EVLWI_BSA_**	370±155	329; 0.42	410±140	400; 0.34	322±132	283.8; 0.41	361±171^‡^	298.7; 0.47	415±178	372.1; 0.43
**EVLWI_height_**	4.20±2.02	3.75; 0.48	4.19±1.50	4.06; 0.36	3.60±1.51	3.17; 0.42	4.6±2.6^§^	3.84; 0.57	4.52±2.00	4.00; 0.44
**EVLWI_TLC_**	120±56	102; 0.47	119±43	111; 0.36	103±47	88; 0.46	132±69^§^	113; 0.52	124±62	110; 0.50
**PaO_2_**	92.9±20.7	90.9; 0.22	94.5±20.1	91.6; 0.21	86.5±16.7	83.0; 0.19	95.1±22.1	96.0; 0.23	121.7±70	102; 0.58
**FiO_2_**	0.47±0.16	0.45; 0.34	0.44±0.14	0.40; 0.32	0.51±0.17	0.50; 0.33	0.46±0.16	0.40; 0.35	59.6±24.9	50; 0.42
**P_aw_**	13.5±4.4	13.0; 0.33	12.4±3.4	12.0; 0.27	13.5±4.4	13.0; 0.33	14.7±5.1	13.0; 0.35	15.7±4.3	15.6; 0.27
**PaO_2_/F_i_O_2_**	219±82	211; 0.37	230±70	226; 0.30	182±76	170; 0.42	188±71	225; 0.38	242±115	229; 0.48
P_aw_*PaO2/FiO2	7.05±5.95	5.9; 0.84	5.7±3.5	5.0; 0.61	8.1±5.9	7.0; 0.73	7.8±7.5	6.0; 0.96	8.8±0.64	6.8; 0.73
**PEEP**	8.6±3.0	8.0; 0.35	7.9±2.6	8.0; 0.33	8.9±3.3	8.0;0.37	9.1±3.0	8.0; 0.33	8.8±3.4	9.0; 0.39
**Measurements**	693		263		167		263		1426	

*Intra*-group comparison † p<0.001 vs. EVLWI_act_ (for EVLWI_pred_, EVLWI_id_ and EVLWI_adj_) within the same BMI-group.

*Inter*-group comparison ‡ p<0.001 group with BMI≥30 kg/m^2^ vs. group with normal BMI (BMI<25 kg/m^2^).

(BMI≥30 kg/m^2^ vs. BMI<25 kg/m^2^) § p>0.05 group with BMI≥30 kg/m^2^ vs. group with normal BMI (BMI<25 kg/m^2^).

1. **Weight and weight-correction formula based indexations.** To compare the distribution of normal (EVLWI<7 ml/kg), slightly elevated (7≤EVLWI<10 ml/kg) and markedly elevated EVLWI (EVLWI≥10 ml/kg) in dependency of the indexation, un-indexed EVLW was indexed according to actual (EVLWI_act_), predicted (EVLWI_pred_), ideal (EVLWI_id_) and adjusted BW (EVLWI_adj_) using the formulas mentioned in Table 1. The cut-offs of 10 ml/kg and 14 ml/kg have been demonstrated to be associated with ARDS [15] and mortality [5].
  –*Intra-group comparisons*: For the weight-related indexations, intragroup comparisons of weight-correction based EVLWI_pred_, EVLWI_id_ and EVLWI_adj_ to EVLWI_act_ were performed for all patients and the subgroups with BMI<25 kg/m^2^, 25≤BMI<30 kg/m^2^ and BMI≥30 kg/m^2^ (Wilcoxon-test for paired samples; Table 2).
  –*Inter-group comparisons*: Furthermore, we compared EVLWI according to all investigated indexations (also including EVLWI_height_, EVLWI_BMI_, EVLWI_BSA_ and EVLWI_TLC_) between patients with BMI<25 kg/m^2^ and patients with BMI≥30 kg/m^2^ (Wilcoxon-test for unpaired samples; Table 2).
2. Association of EVLW(I) to PaO_2_/FiO_2_ and oxygenation index (OI =  mean airway pressure * F_i_O_2_ * PaO_2_^−1^). For appropriate analysis of multiple serial measurements in 231 patients from the two groups several statistical analyses were performed:
  2a) *Prediction of critical thresholds of PaO_2_/FiO_2_ and oxygenation index*: The clinically relevant prediction of critical thresholds of “PaO_2_/FiO_2_<200 mmHg” (primary endpoint) and “OI>10” by EVLW(I) was investigated using receiver operating characteristics area under the curve (ROC-AUC) analyses of all measurements.
  2b) *Inter-individual (“between-subject”)* correlations: Furthermore, correlations of EVLW and differently indexed EVLWI to PaO_2_/FiO_2_ and oxygenation index (OI =  mean airway pressure * F_i_O_2_ * PaO_2_^−1^) were calculated. Since multiple serial measurements within 241 different patients were available, we analysed *inter-* and *intra*-individual correlations.
  To correct for different numbers of measurements for each patient, the means of EVLW(I), PaO_2_/FiO_2_ and OI were calculated for each individual patient (“one point per patient”). Subsequently the correlations between EVLW(I) and PaO_2_/FiO_2_ and between EVLWI and OI were calculated.
  2c) *Intra-individual (“within subject”) correlations*: The above-mentioned “one point per patient” analyses reflect the *inter-individual* association of EVLW(I) to PaO_2_/FiO_2_ and OI. However, in cases of multiple serial measurements within different patients, *between-subject heterogeneity* may obscure correlations *on an individual level (within subject correlation)* which might be even more interesting than the inter-individual association. The effect of the confounder (between-subject heterogeneity) can be removed by calculating “partial” correlation between EVLW(I) and PaO_2_/FiO_2_ (or OI) adjusting for heterogeneity of different patients (individual patient number/identifier as the adjustment factor).
3. **Mortality analysis.** Better prediction of mortality by EVLWI according to any indexation might be related to the direct association of the indexation to mortality. To overcome this problem, multiple binary logistic regression analysis regarding mortality included the first and last values of *unindexed* EVLW as well as BW_act_, height, gender and acute physiology and chronic health evaluation (version 2, APACHE-II).

All analyses were performed separately in both groups and in the merged data, with the only exceptions being intergroup-comparisons and mortality analyses which were restricted to the BMI-representative Munich-evaluation-group. Results of merged data were considered superior to those derived from sub-groups. No correction of p-values was applied to adjust for multiple testing. However, results of all statistical tests being conducted were thoroughly reported so that an informal adjustment of p-values can be performed while reviewing the data [27].

All statistical tests were conducted 2-sided and a p-value <0.05 was considered to indicate statistical significance. The software used was IBM SPSS statistics, version 20.

## Results

### Patients' characteristics

A total of 2119 TPTDs (each with triplicate TPTD) were performed in 231 patients from both groups.

Table 2 summarizes the patients' characteristics of both groups.

1 **Weight and weight-correction formula based indexations.** *Comparison of EVLWI_act_, EVLWI_pred_, EVLWI_id_ and EVLWI_adj_*_:_ Table 2 demonstrates that in the Munich-evaluation-group mean values of EVLWI_pred_, EVLWI_adj_ and EVLWI_id_ were significantly *higher* than EVLWI_act_ in the subgroups of patients with BMI≥30 kg/m^2^ and with 25≤BMI<30 kg/m^2^ as well as for the totality of patients (*intra-*BMI-group-comparison). By contrast, EVLWI_pred_ and EVLWI_adj_ were significantly *lower* than EVLWI_act_ in the subgroup of patients with a *normal* BMI.

Similarly, in the Antwerp-validation-group mean values of EVLWI_pred_, EVLWI_adj_ and EVLWI_id_ were higher than mean the EVLWI_act_.

**Figure 1:**
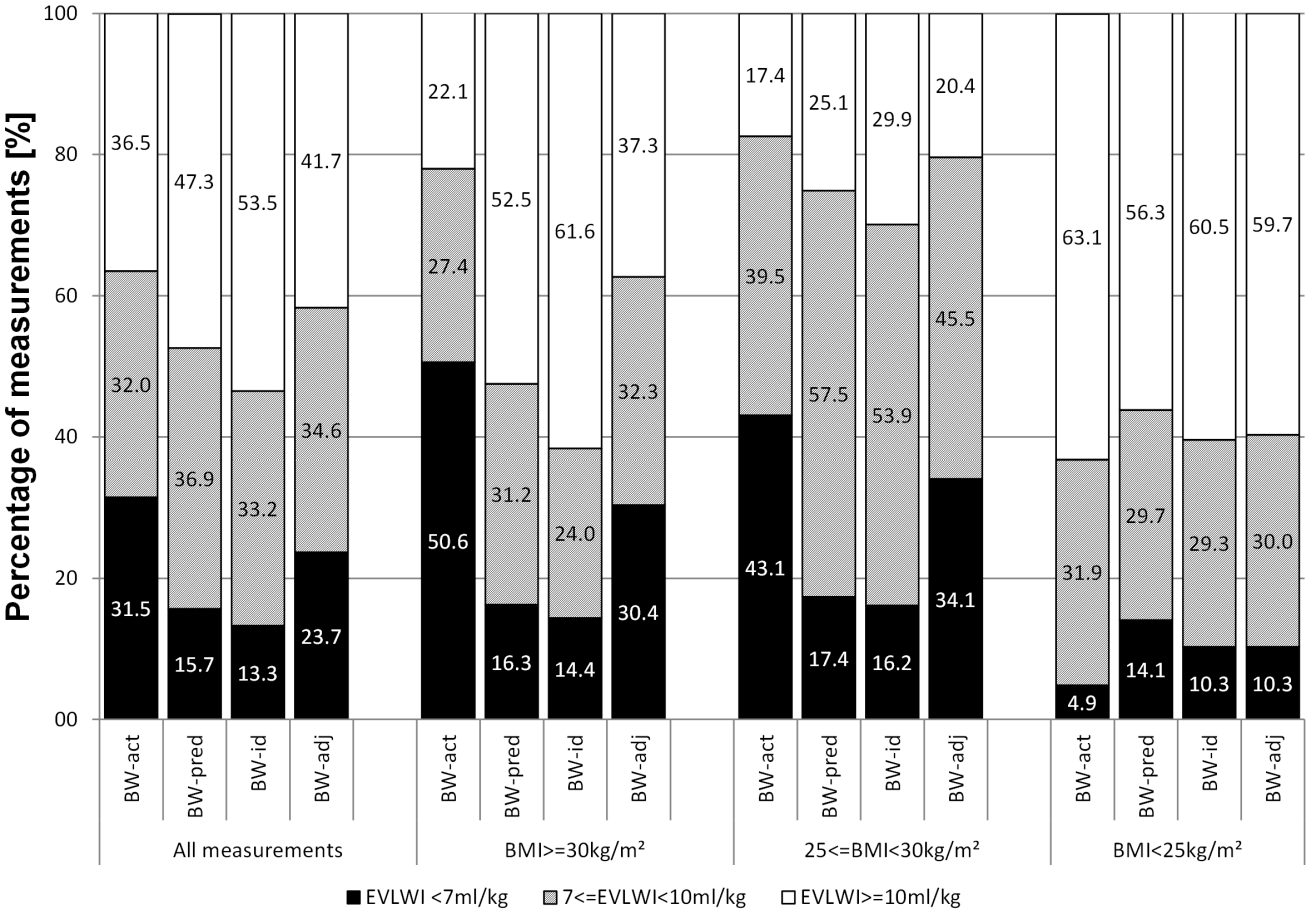
Distribution of measurements classified as “normal” (EVLWI<7 mL/kg), “moderately elevated” (7≤EVLWI<10 mL/kg) and “markedly elevated” (EVLWI≥10 mL/kg) depending on the indexation of EVLWI according to weight-based indexations to BW_act_, BW_pred_, BW_id_ and BW_adj_. Numbers in the columns indicate the percentage of measurements within this classification (*p<0.05 and ** p<0.001 vs. percentage of normal measurements of EVLWI_act_<7 mL/kg within the same BMI-category).

*Impact of indexation according to different weight correction-formulas for the classification of EVLWI*: Distribution of EVLWI-values classified as normal (EVLWI<7 ml/kg), moderately elevated (7 ml/kg≤EVLWI<10 ml/kg) and markedly elevated EVLWI (EVLWI≥10 ml/kg) significantly varied among the patients with a BMI≥30 kg/m^2^ as well as in the total patient groups depending on the weight used for indexation of EVLWI (Fig. 1). For example in patients with a BMI≥30 kg/m^2^, 51% (133/263) of EVLWI_act_ measurements were within the normal range (EVLWI<7 ml/kg). By contrast only 16% (43/263; p<0.001 vs. EVLWI_act_), 14%, (38/263; p<0.001) and 30% of the measurements (79/263; p<0.001) were within the normal range if EVLWI was indexed according to predicted, ideal and adjusted BW, respectively. In addition to the different distributions of EVLWI classifications, different indexations obviously had an impact on the coefficient of variation (COV), in particular in patients with a BMI≥30 kg/m^2^ (Table 2). COV amongst these patients was markedly lower for EVLWI_act_ (0.41) than for EVLWI_height_ (0.57) or un-indexed EVLW (0.60). *Inter*-BMI-group-comparison demonstrated significantly lower values in patients with a BMI≥30 kg/m^2^ for EVLWI_act_, EVLWI_BMI_, EVLWI_BSA_ and EVLWI_adj_, whereas there was no inter-group difference for EVLW, EVLWI_pred_, EVLWI_id_, EVLWI_height_ and EVLWI_TLC_. In conclusion, significant inter-BMI-group-differences were found only for EVLWI_act_ and indexations including BW_act_ in their formulas (BW_adj_, BMI and BSA) (Table 2).

2 **Association of EVLW(I) to parameters of pulmonary function.** *2a.) Prediction of “PaO_2_/FiO_2_<200 mmHg” (primary endpoint) and “OI>10”*: As demonstrated in Table 3, in the Munich-evaluation-group the greatest ROC-AUCs regarding *“PaO_2_/FiO_2_<200 mmHg”* were found for un-indexed EVLW (ROC-AUC 0.758; 95%-CI: 0.637–0.880) and EVLWI_height_ (ROC-AUC 0.746; 95%-CI: 0.622–0.869). EVLWI_act_ provided the lowest ROC-AUC (0.685, 95%-CI: 0.554–0.817)

**Table 3:** Comparison of receiver operating characteristics area under the curve (ROC-AUC) regarding „PaO_2_/FiO_2_<200“ and “Oxygenation-Index (OI)>10” depending on indexation of extravascular lung water (index) EVLW(I): ROC-AUCs for different indexations of EVLW(I) regarding „PaO_2_/FiO_2_<200“ (left side of the table) and “OI>10” (right side of the table).

“ROC-AUCs regarding PaO_2_/FiO_2_<200 mmHg”				p-values for comparison of ROC-AUCs									ROC-AUCs regarding “OI>10”		
**Patients**		AUC	95%-CI	EVLW	EVLWI_act_	EVLWI_pred_	EVLWI_id_	EVLWI_adj_	EVLW_BMI_	EVLWI_BSA_	EVLWI_height_	EVLWI_TLC_		AUC	95%-CI
**Total**	EVLW	0.728	0.673–0.783		0.055	0.331	0.336	0.183	0.034*	0.377	0.215	0.304	EVLW	0.756	0.695–0.817
**Total**	EVLWI_act_	0.683	0.626–0.741	0.017*		0.104	0.094	0.054	0.382	0.001*	0.017*	0.157	EVLWI_act_	0.716	0.649–0.782
**Total**	EVLWI_pred_	0.707	0.650–0.763	0.085	0.158		0.386	0.263	0.160	0.370	0.138	0.319	EVLWI_pred_	0.748	0.686–0.810
**Total**	EVLWI_id_	0.707	0.650–0.763	0.069	0.155	0.399		0.235	0.145	0.377	0.128	0.295	EVLWI_id_	0.749	0.686–0.811
**Total**	EVLWI_adj_	0.699	0.643–0.756	0.027*	0.109	0.284	0.279		0.141	0.070	0.050*	0.353	EVLWI_adj_	0.739	0.675–0.802
**Total**	EVLW_BMI_	0.693	0.636–0.750	0.021*	0.227	0.227	0.300	0.343		0.012*	0.019*	0.216	EVLW_BMI_	0.719	0.653–0.785
**Total**	EVLWI_BSA_	0.718	0.663–0.774	0.249	0.000*	0.215	0.201	0.005*	0.026*		0.198	0.342	EVLWI_BSA_	0.752	0.691–0.814
**Total**	EVLWI_height_	0.729	0.674–0.784	0.388	0.007*	0.015*	0.008*	0.005*	0.024*	0.137		0.136	EVLWI_height_	0.762	0.702–0.822
**Total**	EVLWI_TLC_	0.706	0.650–0.763	0.116	0.187	0.396	0.397	0.327	0.321	0.245	0.038*		EVLWI_TLC_	0.745	0.682–0.807
				EVLW	EVLWI_act_	EVLWI_pred_	EVLWI_id_	EVLWI_adj_	EVLW_BMI_	EVLWI_BSA_	EVLWI_height_	EVLWI_TLC_			
**Munich**	EVLW	0.758	0.637–0.880		0.180	0.398	0.398	0.352	0.184	0.323	0.370	0.397	EVLW	0.732	0.583–0.881
**Munich**	EVLWI_act_	0.685	0.554–0.817	0.076		0.184	0.175	0.101	0.351	0.109	0.135	0.169	EVLWI_act_	0.669	0.502–0.835
**Munich**	EVLWI_pred_	0.713	0.585–0.841	0.131	0.313		0.399	0.335	0.273	0.354	0.382	0.325	EVLWI_pred_	0.729	0.580–0.879
**Munich**	EVLWI_id_	0.710	0.581–0.839	0.091	0.328	0.373		0.324	0.264	0.345	0.379	0.350	EVLWI_id_	0.730	0.580–0.880
**Munich**	EVLWI_adj_	0.711	0.582–0.840	0.114	0.200	0.397	0.398		0.269	0.396	0.285	0.297	EVLWI_adj_	0.714	0.560–0.869
**Munich**	EVLW_BMI_	0.716	0.588–0.844	0.168	0.114	0.398	0.395	0.394		0.213	0.170	0.257	EVLW_BMI_	0.681	0.517–0.845
**Munich**	EVLWI_BSA_	0.728	0.602–0.855	0.137	0.072	0.319	0.272	0.156	0.349		0.213	0.327	EVLWI_BSA_	0.716	0.562–0.870
**Munich**	EVLWI_height_	0.746	0.622–0.869	0.180	0.107	0.130	0.070	0.129	0.271	0.212		0.399	EVLWI_height_	0.737	0.589–0.885
**Munich**	EVLWI_TLC_	0.707	0.577–0.836	0.123	0.351	0.305	0.382	0.392	0.392	0.285	0.121		EVLWI_TLC_	0.736	0.588–0.884
				EVLW	EVLWI_act_	EVLWI_pred_	EVLWI_id_	EVLWI_adj_	EVLW_BMI_	EVLWI_BSA_	EVLWI_height_	EVLWI_TLC_			
**Antwerp**	EVLW	0.725	0.664–0.787		0.147	0.225	0.228	0.164	0.077	0.390	0.187	0.173	EVLW	0.771	0.705–0.836
**Antwerp**	EVLWI_act_	0.710	0.648–0.773	0.301		0.296	0.286	0.256	0.395	0.003*	0.060	0.359	EVLWI_act_	0.739	0.669–0.810
**Antwerp**	EVLWI_pred_	0.723	0.661–0.784	0.390	0.326		0.294	0.366	0.290	0.173	0.036*	0.110	EVLWI_pred_	0.756	0.688–0.824
**Antwerp**	EVLWI_id_	0.724	0.662–0.785	0.395	0.315	0.225		0.353	0.280	0.177	0.033*	0.126	EVLWI_id_	0.757	0.689–0.825
**Antwerp**	EVLWI_adj_	0.722	0.660–0.783	0.383	0.250	0.397	0.391		0.251	0.013*	0.035*	0.394	EVLWI_adj_	0.752	0.683–0.821
**Antwerp**	EVLW_BMI_	0.704	0.641–0.767	0.173	0.337	0.268	0.254	0.165		0.016*	0.044*	0.353	EVLW_BMI_	0.738	0.667–0.809
**Antwerp**	EVLWI_BSA_	0.734	0.673–0.795	0.307	0.022*	0.264	0.275	0.100	0.024*		0.356	0.126	EVLWI_BSA_	0.773	0.708–0.839
**Antwerp**	EVLWI_height_	0.735	0.674–0.796	0.074	0.151	0.151	0.156	0.176	0.078	0.393		0.033*	EVLWI_height_	0.778	0.713–0.842
**Antwerp**	EVLWI_TLC_	0.716	0.654–0.778	0.329	0.383	0.069	0.079	0.353	0.345	0.183	0.098		EVLWI_TLC_	0.750	0.681–0.819
				EVLW	EVLWI_act_	EVLWI_pred_	EVLWI_id_	EVLWI_adj_	EVLW_BMI_	EVLWI_BSA_	EVLWI_height_				

*p<0.05.

In the middle of the table p-values for the comparison of ROC-AUCs according to different indexations are given.

In general, these observations were confirmed in the Antwerp-validation-group: EVLWI_height_ had the highest predictive capability (ROC-AUC 0.735; 95%-CI: 0.674–0.796), whereas weight-indexed EVLWI_act_ (ROC-AUC 0.710; 95%-CI: 0.648–0.773) and EVLWI_BMI_ (ROC-AUC 0.704; 95%-CI: 0.641–0.767) provided the smallest ROC-AUCs.

Statistical analysis of the merged data of both groups demonstrated a number of significant differences between different indexations, summarized as follows:

1 The greatest ROC-AUC regarding *“PaO_2_/FiO_2_ <200 mmHg”* was found for EVLWI_height_ (ROC-AUC 0.729; 95%-CI: 0.674–0.784; primary endpoint)
2 The ROC-AUC was significantly greater for EVLWI_height_ compared to all other indexations including EVLWI_act_ (ROC-AUC 0.683; 95%-CI: 0.626–0.741; p = 0.007) and EVLWI_pred_ (ROC-AUC 0.707; 95%-CI: 0.650–0.763; p = 0.015). Only un-indexed EVLW (ROC-AUC 0.728; 95%-CI: 0.673–0.783) and EVLWI_BSA_ (ROC-AUC 0.718; 95%-CI: 0.663–0.774; p = 0.137) were not significantly inferior compared to EVLW_height_.

Regarding the prediction of the threshold “OI>10”, in both collectives as well as in the merged data, the largest ROC-AUCs were obtained for EVLWI_height_ and EVLW, with the lowest for EVLWI_act_ (Table 4): 0.737 (0.589–0.885), 0.732 (0.583–0.881) and 0.669 (0.502–0.835) in the Munich-evaluation-group, 0.778 (0.713–0.842), 0.771 (0.705–0.836), and 0.739 (0.669–0.810) in the Antwerp-validation group and 0.762 (0.702–0.822), 0.756 (0.695–0.817) and 0.716 (0.649–0.782) for the merged data, respectively.

**Table 4:** Intra- and inter-individual correlations of extravascular lung water (index) EVLW(I) to PaO_2_/FiO_2_.

		Comparison to PaO_2_/FiO_2_				Comparison to oxygenation index(mean airway pressure* FiO_2_*/PaO_2_)			
Group	Parameter	correlation of patient means (between-subject correlation; Spearman)		partial correlation: all measurements (within-subject correlation)		correlation of patient means (between-subject correlation; Spearman)		partial correlation: all measurements (within-subject correlation)	
		r-value	r^2^-value	r-value	r^2^-value	r-value	r^2^-value	r-value	r^2^-value
Munich	EVLW	−0.34*	0.12*	−0.74**	0.55**	0.44*	0.19**	0.80**	0.64**
Munich	EVLWI_act_	−0.21	0.04	−0.66**	0.44**	0.32*	0.10**	0.57**	0.32**
Munich	EVLWI_pred_	−0.27	0.07	−0.69**	0.48**	0.47*	0.22**	0.76**	0.58**
Munich	EVLWI_ideal_	−0.26	0.07	−0.69**	0.48**	0.48**	0.23**	0.76**	0.58**
Munich	EVLWI_adj_	−0.29*	0.08*	−0.68**	0.46**	0.46*	0.21**	0.68**	0.46**
Munich	EVLW_BMI_	−0.26	0.07	−0.72**	0.52**	0.31*	0.10**	0.65**	0.42**
Munich	EVLWI_BSA_	−0.28	0.08	−0.71**	0.50**	0.42*	0.18**	0.71**	0.50**
Munich	EVLWI_height_	−0.32*	0.10*	−0.73**	0.53**	0.45*	0.20**	0.78**	0.61**
Munich	EVLWI_TLC_	−0.26	0.07	−0.69**	0.48**	0.48**	0.23**	0.76**	0.58**
Antwerp	EVLW	−0.43**	0.18**	−0.68**	0.46**	0.45**	0.20**	0.76**	0.58**
Antwerp	EVLWI_act_	−0.42**	0.18**	−0.63**	0.40**	0.40**	0.16**	0.72**	0.52**
Antwerp	EVLWI_pred_	−0.43**	0.18**	−0.65**	0.42**	0.44**	0.19**	0.73**	0.53**
Antwerp	EVLWI_ideal_	−0.43**	0.18**	−0.65**	0.42**	0.44**	0.19**	0.73**	0.53**
Antwerp	EVLWI_adj_	−0.43**	0.18**	−0.62**	0.38**	0.43**	0.18**	0.70**	0.49**
Antwerp	EVLW_BMI_	−0.38**	0.14**	−0.62**	0.38**	0.37**	0.14**	0.70**	0.49**
Antwerp	EVLWI_BSA_	−0.46**	0.21**	−0.66**	0.44**	0.46**	0.21**	0.75**	0.56**
Antwerp	EVLWI_height_	−0.45**	0.20**	−0.68**	0.46**	0.47**	0.22**	0.76**	0.58**
Antwerp	EVLWI_TLC_	−0.41**	0.17**	−0.63**	0.40**	0.42**	0.18**	0.72**	0.52**
All Patients	EVLW	−0.39**	0.15**	−0.68**	0.46**	0.46**	0.21**	0.78**	0.61**
All Patients	EVLWI_act_	−0.36**	0.13**	−0.60**	0.36**	0.39**	0.15**	0.69**	0.48**
All Patients	EVLWI_pred_	−0.38**	0.14**	−0.63**	0.40**	0.46**	0.21**	0.75**	0.56**
All Patients	EVLWI_ideal_	−0.37**	0.14**	−0.60**	0.36**	0.47**	0.22**	0.75**	0.56**
All Patients	EVLWI_adj_	−0.38**	0.14**	−0.63**	0.40**	0.44**	0.19**	0.71**	0.50**
All Patients	EVLW_BMI_	−0.34**	0.12**	−0.61**	0.37**	0.38**	0.14**	0.70**	0.49**
All Patients	EVLWI_BSA_	−0.41**	0.17**	−0.65**	0.42**	0.47**	0.22**	0.74**	0.55**
All Patients	EVLWI_height_	−0.41**	0.17**	−0.67**	0.45**	0.48**	0.23**	0.77**	0.59**
All Patients	EVLWI_TLC_	−0.38**	0.14**	−0.63**	0.40**	0.44**	0.19**	0.74**	0.55**

Partial correlations were calculated with the individual patient as the controlling adjustment variable. *p<0.05 **p<0.001.

For the merged data, the AUC was significantly higher for EVLWI_height_ (0.762) compared to EVLWI_act_ (0.716; p = 0.017), EVLWI_adj_ (0.739; p = 0.05) and EVLWI_BMI_ (0.719; p = 0.019).

*2b.), 2.c) Inter-individual and intra-individual correlations of EVLW(I)to PaO_2_/FiO_2_ and to oxygenation index*: In general, the *inter*-individual association (represented by patients' means) of EVLW(I) to PaO_2_/FiO_2_ and OI was not as strong as the *intra*-individual association represented by partial correlations. Means of EVLW(I) and PaO_2_/FiO_2_ moderately correlated with r-values between −0.21 and −0.46. By contrast, *intra*-individual correlations represented by partial correlations provided r-values between −0.6 and −0.74 (Table 4).

Within the Munich-evaluation-group only the patients' means of EVLW, EVLWI_height_ and EVLWI_adj_ significantly correlated with PaO_2_/FiO_2_ (r = −0.34; p = 0.017, r = −0.32, p = 0.026 and r = −0.29; p = 0.041, respectively). In contrast, the patients' mean EVLWI according to all other indexations including EVLWI_pred_ and EVLWI_act_ did not correlate to PaO_2_/FiO_2_. Overall, the lowest r-values were found for EVLWI_act_. The highest coefficients of correlations to PaO_2_/FiO_2_ were found for un-indexed EVLW and EVLWI_height_ (Table 4).

However, in the same group all *partial* correlations of EVLW(I) and PaO_2_/FiO_2_ were highly significant (p<0.001) and provided coefficients of partial correlation between −0.66 and −0.74.

Similar data were obtained for the correlation between OI and EVLW and its indexations (Table 3). Intra-individual partial correlations with r-values between 0.57 and 0.80 were more pronounced than inter-individual correlations represented by r-values between 0.31 and 0.48 for correlations of patients' means. All these correlations were significant. In both groups as well as in the merged data the highest coefficients of correlation to OI were found for EVLWI_height_ and EVLW, the lowest for EVLWI_act_ (r-values for merged data 0.77, 0.78 and 0.69, respectively) (Table 4; partial correlation).

3 **Mortality analysis.** Univariable logistic regression analysis of the Munich-evaluation-group demonstrated a significant association between mortality with APACHE-II-Score (p = 0.005; 95%-CI: 1.063–1.422; β-coefficient of regression 0.207), but not with age (p = 0.262), height (p = 0.265) and BW_act_ (p = 0.123). The strongest association to mortality was found for the *last* EVLW (p = 0.001; 95%-CI: 1.185–2.035; β-coefficient of regression 0.440 for increments in EVLW of 100 ml). The *first* EVLW was associated with mortality with borderline significance (p = 0.054; 95%-CI: 0.098–1.350; β-coefficient of regression 0.149).

Subsequently, a multivariable logistic regression analysis regarding mortality was performed. A model including APACHE-II, the first and the last EVLW provided high predictive capabilities regarding mortality (Nagelkerkes R^2^ = 0.697). First (p = 0.021) and last (p = 0.004) EVLW were independently associated to mortality. The APACHE-II score slightly failed to reach significance (p = 0.064), but markedly contributed to the R^2^-value of the total model (R^2^ = 0.628 without APACHE-II).

**Figure 2:**
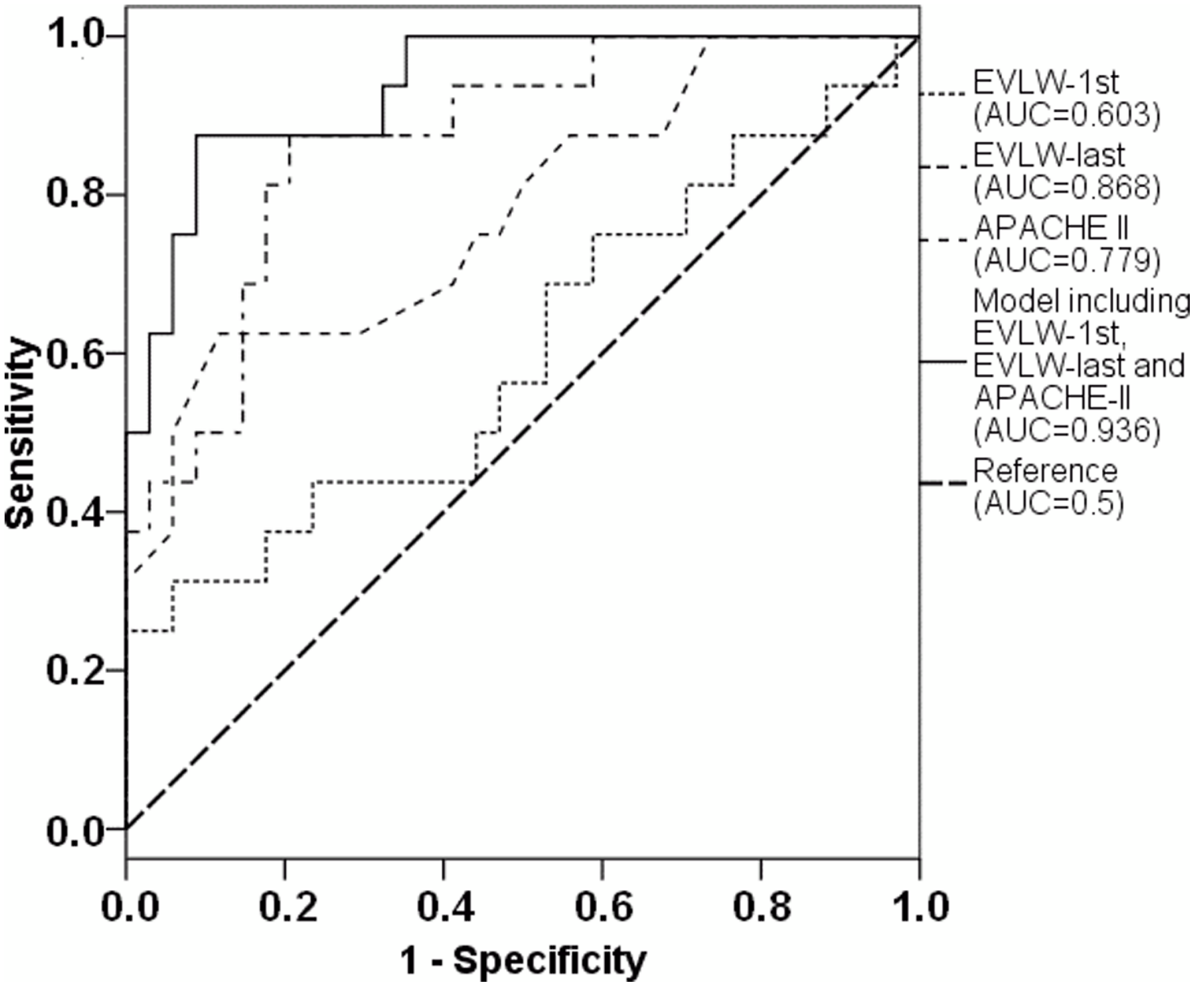
ROC curve regarding the prediction of mortality provided by first EVLW, last EVLW, APACHE-II and regression model combining first EVLW, last EVLW and APACHE-II.

ROC-analysis (Figure 2) regarding mortality demonstrated high predictive capabilities of the model including APACHE-II as well as first and last EVLW (AUC 0.936; 95%-CI: 0.868–1.000; p<0.001), which provided a markedly larger ROC-AUC than each of the included single parameters. Among single parameters, the last EVLW (AUC 0.868; 95%-CI: 0.765–0.970; p<0.001) and APACHE-II (AUC 0.779; 95%-CI: 0.636–0.923; p = 0.002) provided high predictive capabilities compared to the first EVLW (AUC 0.603; 95%-CI: 0.424–0.782; p = 0.244).

## Discussion

Data regarding EVLW-indexation are contradictory. Therefore, our study investigated the association between different indexations of EVLW(I) to PaO_2_/FiO_2_ and OI in two groups with mechanical ventilation and representative distribution of BMI. Our study demonstrated that

1. indexation of EVLWI to BW_act_ is inferior to no indexation at all,
2. indexation to BW_pred_ might provide a certain improvement compared to indexation to BW_act_ and
3. indexation according to height or no indexation at all (EVLW) are superior to indexation to BW_act_ or BW_pred_.

These results are – at first glance – surprising, as several studies have suggested BW_pred_ as the appropriate indexation factor [12]–[14].

Historically, different techniques have been established to quantify pulmonary edema termed as “EVLW” which was originally determined without indexation. Early studies frequently used animal models with post-mortem gravimetric determination of EVLW as the gold-standard [1], [ 2], [ 4], [ 10]. Regarding investigations in different species, indexation to BW_act_ provided “basic” indexation allowing *interspecies* comparisons between different animal and human data.

However, in obese patients indexation to BW_act_ might inappropriately diminish indexed EVLWI_act_. Based on a better prediction of *mortality* rather than on a better correlation to PaO_2_/FiO_2_, superiority of EVLWI_pred_ to EVLWI_act_ has been suggested: In the study by Phillips et al. [12] including 19 patients, EVLWI_act_ was not related to mortality. By contrast, mortality of the seven out of 19 patients was univariately associated to EVLWI_pred_. However, EVLWI_act_ obviously provided a better correlation to PaO_2_/FiO_2_ than EVLWI_pred_ (coefficient of correlation: −0.525 for EVLWI_pred_ and −0.773 for EVLWI_act_). With only 19 patients included, this study did not approach a multivariate mortality-analysis. Another study that included 44 patients (225 measurements) demonstrated better discrimination of ARDS- and non-ARDS-patients by EVLWI_pred_ compared to EVLWI_act_ [13]. A third study (44 patients; 44 measurements) showed the improved association of mortality by EVLWI_pred_ compared to EVLWI_act_ in a multivariate model [14]. However, similarly to the data of Phillips et al. this study did not demonstrate a better correlation of EVLWI_pred_ to PaO_2_/FiO_2_ compared to EVLWI_act_ (coefficients of correlation −0.57 vs. −0.55). This is in accordance with two more recent studies suggesting a comparable [16] or even stronger [15] association of EVLWI_act_ compared to EVLWI_pred_ with mortality.

Nevertheless, mortality is multifactorial, also depending on “do-not-resuscitate” statements and might be directly associated to some of the components of indexation (weight, BMI) [28]-[36]. E.g. further analysis of the data by Phillips et al. demonstrates that mean EVLW and BMI were increased to a similar degree in the non-survivors compared to survivors (45% and 31%, respectively). Therefore, mortality is not an obvious endpoint to compare the appropriateness of different indexations of EVLWI, particularly when applied in small mono-centric collectives.

In our study-groups un-indexed EVLW - next to EVLWI_height_ - provided the highest predictive capability regarding PaO_2_/FiO_2_ and OI. This indicates that particularly *weight*-based indexation might be a confounder rather than an improvement of the inter-individual comparison of EVLW(I) in an adult population. In the analysis of patients' means, PaO_2_/FiO_2_ was significantly associated to unindexed EVLW, EVLWI_height_ and EVLWI_adj_, whereas the correlation was not significant for all other indexations. These findings suggest that the association of EVLW(I) to PaO_2_/FiO_2_ might be obscured by inappropriate indexation. In general, the association of EVLW(I) was closer to OI compared to PaO_2_/FiO_2_. Including mean airway pressure in addition to PaO_2_/FiO_2_, OI also reflects the Positive End Expiratory Pressure (PEEP), peek and plateau pressure, ventilation mode (there is a usually higher mean airway pressure in controlled compared to assisted ventilation) as well as I:E ratio. Since the association of EVLW(I) to OI was not extensively investigated in the previous studies, the close association in our study might even strengthen the role of EVLW(I) as a parameter of pulmonary (patho)physiology.

With regard to indexation of other pulmonary parameters, the strong performance of height as an indexation for EVLWI is not surprising. As stated in recent consensus guidelines “lung volumes are related to body size, with standing height being the most important factor” [21].

Furthermore, a look at the “weight correction-formulas” demonstrates that BW_pred_ and BW_id_ (Table 1) do not contain any weight at all, but simply adjust height for gender and subtract a length-constant [20], [ 21], [ 28].

In addition to weight and height, the third major determinant of most indexation formulas is *gender*, which has impact on BW_pred_, BW_id_, BW_adj_ and TLC. With regard to the above-mentioned formulas, indexation according to BW_id_ increases EVLWI by 5.5% for women. EVLWI_pred_ of women with a height between 150 and 190 cm is increased by 5–10% compared to men with the same height. However, this marked impact of gender on EVLW is not substantiated by our data: Multiple regression analysis regarding EVLW in our merged data including the variables age, height, weight, PaO_2_/FiO_2_ and gender demonstrated that gender was not independently associated to EVLW.

Finally, the question remains, whether in adults “no indexation at all” is the answer. Regarding our data in two adult groups with a high variability in body weight and BMI, but lower variability in height, this might be a reasonable option. However, it must be kept in mind that the variability in height was low in these groups: e.g. mean height in the Munich-evaluation-group was 170±10 cm (median 171 cm, range 150–190 cm).

On the other hand it is self-evident that indexation will improve the predictive capabilities in a group with a higher variability of parameters closely associated to EVLW such as height. There is elaborate data on the pulmonary function parameters in children and adolescents: Normal values in these groups with high variability in height and weight are mainly adjusted to height [20], [ 21].

### Limitations of the study

Despite the inclusion of two different groups and the large sample size compared to the previous data, our study has several limitations. Our Munich-evaluation-group was a preselected group of non-operative mechanically ventilated patients with a prolonged ICU-stay. Although this drawback might be - at least in part - outweighed by a re-evaluation in a large group of non-selected anesthesiology patients, the data of both groups might not apply to patients without pulmonary impairment. On the other hand, the significance of modest correlations with r-values as low as -0.29 require cautious interpretation, since large numbers of patients promote significance of modest associations. Furthermore, these data are mainly derived from Caucasians. Despite a “considerable lack of data on lung-volumes in non-Caucasians” [20], [ 37] at least two studies give hints on differences regarding TLC between whites and blacks [38], Polynesians, Northern Indians and Pakistanis [39]–[41]. Finally, we cannot extrapolate our results to a pediatric population in which indexation to height may be much more appropriate than unindexed EVLW which was comparable to EVLW_height_ in our adult groups.

## Conclusions

EVLW is a marker significantly associated to pulmonary function and mortality. Regarding the prediction of PaO_2_/FiO_2_ and OI, indexation of EVLWI_act_ is inappropriate. EVLWI_pred_ provides a slight improvement. The highest predictive capabilities in an adult population were found for EVLWI_height_ and un-indexed EVLW. Therefore, our data suggest that EVLW should be indexed to height (EVLWI_height_) or remain unindexed in adults.
